# Growth factors and growth factor gene therapies for treating chronic wounds

**DOI:** 10.1002/btm2.10642

**Published:** 2023-12-28

**Authors:** James A. Mullin, Erfan Rahmani, Kristi L. Kiick, Millicent O. Sullivan

**Affiliations:** ^1^ Department of Chemical and Biomolecular Engineering University of Delaware Newark Delaware USA; ^2^ Department of Biomedical Engineering University of Delaware Newark Delaware USA; ^3^ Department of Materials Science and Engineering University of Delaware Newark Delaware USA

**Keywords:** chronic wounds, gene therapy, growth factors

## Abstract

Chronic wounds are an unmet clinical need affecting millions of patients globally, and current standards of care fail to consistently promote complete wound closure and prevent recurrence. Disruptions in growth factor signaling, a hallmark of chronic wounds, have led researchers to pursue growth factor therapies as potential supplements to standards of care. Initial studies delivering growth factors in protein form showed promise, with a few formulations reaching clinical trials and one obtaining clinical approval. However, protein‐form growth factors are limited by instability and off‐target effects. Gene therapy offers an alternative approach to deliver growth factors to the chronic wound environment, but safety concerns surrounding gene therapy as well as efficacy challenges in the gene delivery process have prevented clinical translation. Current growth factor delivery and gene therapy approaches have primarily used single growth factor formulations, but recent efforts have aimed to develop multi‐growth factor approaches that are better suited to address growth factor insufficiencies in the chronic wound environment, and these strategies have demonstrated improved efficacy in preclinical studies. This review provides an overview of chronic wound healing, emphasizing the need and potential for growth factor therapies. It includes a summary of current standards of care, recent advances in growth factor, cell‐based, and gene therapy approaches, and future perspectives for multi‐growth factor therapeutics.

**Translational Impact Statement**

Chronic wounds persist as a healthcare challenge despite extensive research on various treatments, including growth factors and gene therapies. Progress in translating these therapeutics to clinical use has been slow, with many growth factor approaches demonstrating promise in preclinical studies but providing limited benefits in clinical trials or clinical application. This review presents recent advances in growth factor therapies and growth factor gene therapies, discusses obstacles to regulatory approval, and offers perspectives on potential innovations for successful clinical translation.

## 1 INTRODUCTION

Chronic wounds, such as diabetic foot ulcers (DFUs), venous leg ulcers (VLUs), and pressure ulcers (PUs), cause an enormous physical and financial burden to both patients and the global healthcare system due to their tendency to remain open for an extended period of time and failure to progress through the normal phases of wound healing.^1^, ^2^ Diabetes mellitus, the underlying cause of DFUs, affects over 530 million individuals worldwide;^3^ patients with diabetes have an estimated 15–25% incidence rate of DFUs over their lifetime, and for those developing DFUs, nearly 70% will not heal within 5 months.^4^, ^5^, ^6^ The extended healing times for DFUs result in poor long‐term outcomes including large numbers of lower leg amputations and high 5‐year mortality rates.^7^, ^8^ Meanwhile, venous insufficiency is the primary cause of VLUs, which make up approximately 80% of the chronic wounds occurring on the lower leg.^9^, ^10^ The incidence rate of VLUs is comparable to the rate for DFUs, with nearly 15% of venous insufficiencies leading to a VLU; however, in contrast with the poor healing rates of DFUs, up to 70% of VLUs will heal within 6 months.^9^, ^11^ The intense care needed for treating all types of chronic wounds leads to annual healthcare costs totaling over $25 billion in United States.^1^ The poor patient outcomes and high treatment costs across all types of chronic wounds indicate a continued need for improved therapeutics.

Chronic wounds develop when the complex, highly coordinated wound healing process, which involves numerous signaling molecules and cellular processes, is disrupted. However, many current clinical approaches fail to treat the underlying physiological causes of wound chronicity. Debridement, the process of removing necrotic tissue and other debris, is widely accepted as the standard of care for chronic wounds, but evidence documenting its effectiveness is limited, and the methods, timing, and extent of debridement vary significantly depending on the patient and practitioner.^12^, ^13^ Furthermore, while debridement can initiate the healing cascade, it is reliant on the subsequent normal progression of healing events for complete wound closure.^14^ Accordingly, many trials for new treatment approaches utilize debridement as one step in the care regime, suggesting that it has an important role in a more comprehensive wound care protocol.^12^ Debridement of the wound is commonly followed by the application of a wound dressing, which is typically a dry cotton gauze or semipermeable film (e.g., Tegaderm).^15^ Other widely used dressings, including hydrogels (e.g., Nu‐Gel), hydrocolloids (e.g., DuoDERM), and foams (e.g., Allevyn), are absorbent and target moisture retention;^15^ however, similar to debridement practices, there is little standardization in the process of choosing a wound dressing, and selection is often reliant on the personal experiences of the clinician.^13^

Alternatively, extracellular matrix (ECM)‐inspired wound dressings with and without cells have gained traction in both the laboratory and the clinic (e.g., Apligraf, Dermagraft, and Omnigraft) as improvements over the current treatment options.^16^ To replicate the structure and function of the ECM, these dressings are generally produced from either decellularized matrix, individual matrix components such as collagen, elastin, or hyaluronic acid, or synthetic materials, all of which provide a bioactive substrate to aid in wound healing by encouraging cell migration and proliferation.^17^ However, the clinical success of these treatments has varied immensely, and many dressings fail to yield even 50% closure rates.^16^, ^18^ Overall, current wound care practices produce inconsistent results, potentially due to shortcomings in addressing all phases of the wound healing cascade and their reliance on innate healing mechanisms to correct aberrant signaling and promote wound closure.

Exogenous growth factor (GF) delivery has emerged as an option for correcting disrupted GF signaling, with Regranex (a topical formulation of platelet‐derived GF (PDGF)) achieving FDA approval, and others, such as Fiblast (fibroblast GF (FGF) formulation), and Heberprot‐P (epidermal GF (EGF)), reaching clinical trials.^19^ However, the instability of GFs in the wound environment as well as safety concerns with the high doses required for efficacy have led to several late‐stage clinical trial failures and challenges with translation.^20^, ^21^ As a result of these limitations, gene delivery systems for endogenous GF production have been of significant interest.^22^ Viral approaches have been explored preclinically and have advanced furthest in early‐stage clinical trials, owing to their higher gene transfer capacity, but safety concerns around immunogenicity and insertional mutagenesis have broadly limited their clinical translation.^23^, ^24^ Nonviral approaches, on the other hand, offer a potentially promising combination of tunability and safety. For chronic wound healing, most nonviral gene therapy strategies tested preclinically are lipid‐ or polymer‐based, but low transfection efficiency has limited their effectiveness.^25^ Moreover, there is a clear need for therapies delivering multiple GFs to correct chronic wounds, but most preclinical and clinical studies have focused on single‐GF therapeutics, limiting therapeutic efficacy.^26^ This review will cover GF‐based therapies for treating chronic wounds including topical application and gene delivery strategies, with a focus on nonviral gene delivery strategies providing controlled expression of one or multiple GFs.

## 2 WOUND PHYSIOLOGY OVERVIEW

### 2.1 Normal wound healing

The skin tissue is typically comprised of three distinct layers—the epidermis, dermis, and hypodermis—which represent the outermost, central, and innermost regions of the tissue, respectively. As the outermost layer of the skin, the epidermis acts as the initial line of defense against the invasion of toxins, endotoxins, infections, and ultraviolet radiation. The keratinocyte and germinal layers also restrict the leakage of tissue fluid and provide antifriction benefits.^27^

The dermis lies directly below the epidermis and consists primarily of ECM components such as collagen and elastin, which provide the skin with its mechanical properties and elasticity, as well as glycoproteins and glycosaminoglycans. A variety of cell types, such as vascular smooth muscle cells, endothelial cells, fibroblasts, and mast cells are also present in this layer. The innermost hypodermis primarily consists of adipose tissue (which serves as an essential shock‐resistant agent and thermal insulator for the body), elastin fibers, and a relatively small quantity of collagen fibers.^28^

The coordinated communication between multiple cell types, GFs, cytokines, and chemokines is necessary for successful wound healing; the sequential steps of wound healing include hemostasis, inflammation, proliferation, and remodeling.

#### 2.1.1 Hemostasis phase

Within a few minutes post‐injury, vascular smooth muscle cells are activated by vasoconstrictors such as endothelin, secreted by damaged endothelium, resulting in constriction of the blood vessels and preventing further bleeding;^29^ however, this process provides only a short‐term remedy, since wound‐induced hypoxia and acidosis result in muscle relaxation and subsequent bleeding. Activation of additional steps in the coagulation cascade is thus necessary to further advance healing. Platelets serve as the key cellular agents involved in the coagulation step. Exposed collagen, von Willebrand factor, and fibronectin at the site of injury bind to glycoprotein IV receptors on the surface of platelets, triggering the activation of platelets.^30^, ^31^ Platelets then release various glycoproteins, facilitating platelet aggregation and binding to ECM. The shape of the platelets shifts from spiky spheres to flattened disks, which allows them to firmly adhere and act as a mechanical seal for injured blood vessels.^32^, ^33^ Once a preliminary plug is created at the injury site, platelets trigger the intrinsic clotting pathway by secreting thrombin, resulting in the formation of a secondary hemostatic plug via the deposition of fibrin meshes. This stable fibrin network serves as matrix for further cellular attachment and proliferation. Various chemotactic factors released by the blood clots, including PDGF, insulin GF (IGF), interleukin (IL)‐1, and transforming GFs (TGF‐α and TGF‐β), attract immune cells from surrounding tissues and the bloodstream to the site of injury, initiating the inflammatory phase of healing.^34^, ^35^

#### 2.1.2 Inflammatory phase

Inflammation initiates instantly upon injury and typically lasts for about 72 h following damage. After early hemostasis, platelet degranulation results in basophil and mast cell activation and the subsequent release of inflammatory mediators such as serotonin, histamine, and proteases, which can cause the formation of intercellular gaps in the endothelium. The expansion of blood vessels and increased vessel permeability promote the efflux of fibrinogen from plasma into the injury site, attracting inflammatory cells such as neutrophils to the wound.^33^, ^36^, ^37^ Typically, the neutrophil count reaches its highest level within 1–2 days following injury, followed by a significant decline after 3 days. Neutrophils are essential to the inflammatory phase because of their role in clearing the wound bed of cellular debris, dead cells, bacteria, and pathogens. Neutrophils follow chemotactic signals to the wound and migrate through the endothelium via rolling, adhesion, and crawling. Upon reaching the site of inflammation, neutrophils employ multiple mechanisms, including the formation of sticky net‐like structures called neutrophil extracellular traps, phagocytosis, and the secretion of reactive oxygen species (ROS), to prevent bacterial invasion.^38^ Monocytes are quickly mobilized from either bone marrow or spleen 2 days post‐injury and differentiate to macrophages and dendritic cells at the wound site via the actions of IL‐4 and tumor necrosis factor (TNF‐α) released by injured cells.^39^ During the initial phase of wound healing, macrophages secrete various cytokines such as IL‐1, IL‐6, and TNF‐α, and play a significant role in forming new blood vessels, controlling inflammatory responses, and driving ECM deposition. Furthermore, macrophages facilitate pathogen clearance and enhance the formation of granulation tissue. Then, 3–4 days after the injury, macrophages begin to secrete GFs and proteins, which attract immune cells to the site of injury and advance the healing process toward the proliferation stage.^33^, ^40^, ^41^, ^42^

#### 2.1.3 Proliferation phase

The proliferation phase starts approximately 4 days after injury and lasts for about 3 weeks. Platelets are responsible for triggering key aspects of this phase through the production of PDGF, FGF, and TGF‐β during hemostasis.^35^ Governed by these signals, fibroblasts, keratinocytes, and other cells in the wound bed work together to form granulation tissue comprised of a loose collagen matrix with hyaluronic acid and fibronectin, new capillaries, and various cell types.^27^ The formation of new blood vessels is essential to ensure the delivery of oxygen, nutrients, and metabolic exchange. In response to a lack of oxygen and cytokines, vascular endothelial GF (VEGF) is released by fibroblasts, macrophages, and keratinocytes, triggering the repair and formation of new blood vessels.^33^ Simultaneously, activated fibroblasts move to the site of injury, resulting in the secretion of glycosaminoglycans such as hyaluronic acid, proteoglycans, fibronectins, and collagen (type ІІІ).^38^ TGF‐β, which is secreted by macrophages and platelets as part of their immune response to injury, can stimulate fibroblast differentiation into myofibroblasts. Myofibroblasts are characterized by increased expression of contractile proteins (such as alpha smooth muscle actin) and enhanced ECM secretion. They also catalyze wound closure through the organization of actin filaments.^43^, ^44^ The process of reepithelialization is facilitated through the release of KGF and EGF by stimulated fibroblasts. Keratinocytes, which are located at the dermal appendages and wound edge, migrate to the wound site, where they cover the wound with a new layer of epidermis.^45^, ^46^ As the wound reepithelialization process nears completion, mechanisms like contact inhibition and downregulation of GFs, such as TGF‐β, trigger the keratinocytes to stop migration. Meanwhile, myofibroblasts undergo apoptosis and the number of capillaries is reduced, which ultimately results in the transition from granulation tissue to an acellular scar.

#### 2.1.4 Remodeling phase

Approximately 3 weeks after injury, the wound maturation and remodeling step begins and may continue for over a year. During this step, all wound‐induced activities slowly decrease and eventually come to a halt. Cells such as macrophages, fibroblasts, and myofibroblasts are no longer needed, and either migrate out of the wound or release regulatory signals such as TNF‐α and IFN‐γ to initiate apoptosis. As the wound enters the remodeling stage, the unstructured type ІІІ collagen within granulation tissue is replaced by type І collagen, which is the primary component of the dermis and is organized in parallel networks.^27^, ^38^, ^44^ This transition increases the scar tissue's tensile strength, which ultimately reaches a maximum of about 70–80% of the tensile strength in intact skin.^47^ Additional changes also occur as endothelial cells and fibroblasts undergoing apoptosis release matrix metalloproteinases (MMPs), which cleave collagen ends. Ultimately, the wound healing process leads to the formation of a fully developed scar with reduced vascularity.^46^, ^48^

### 2.2 Chronic wounds

**FIGURE 1:**
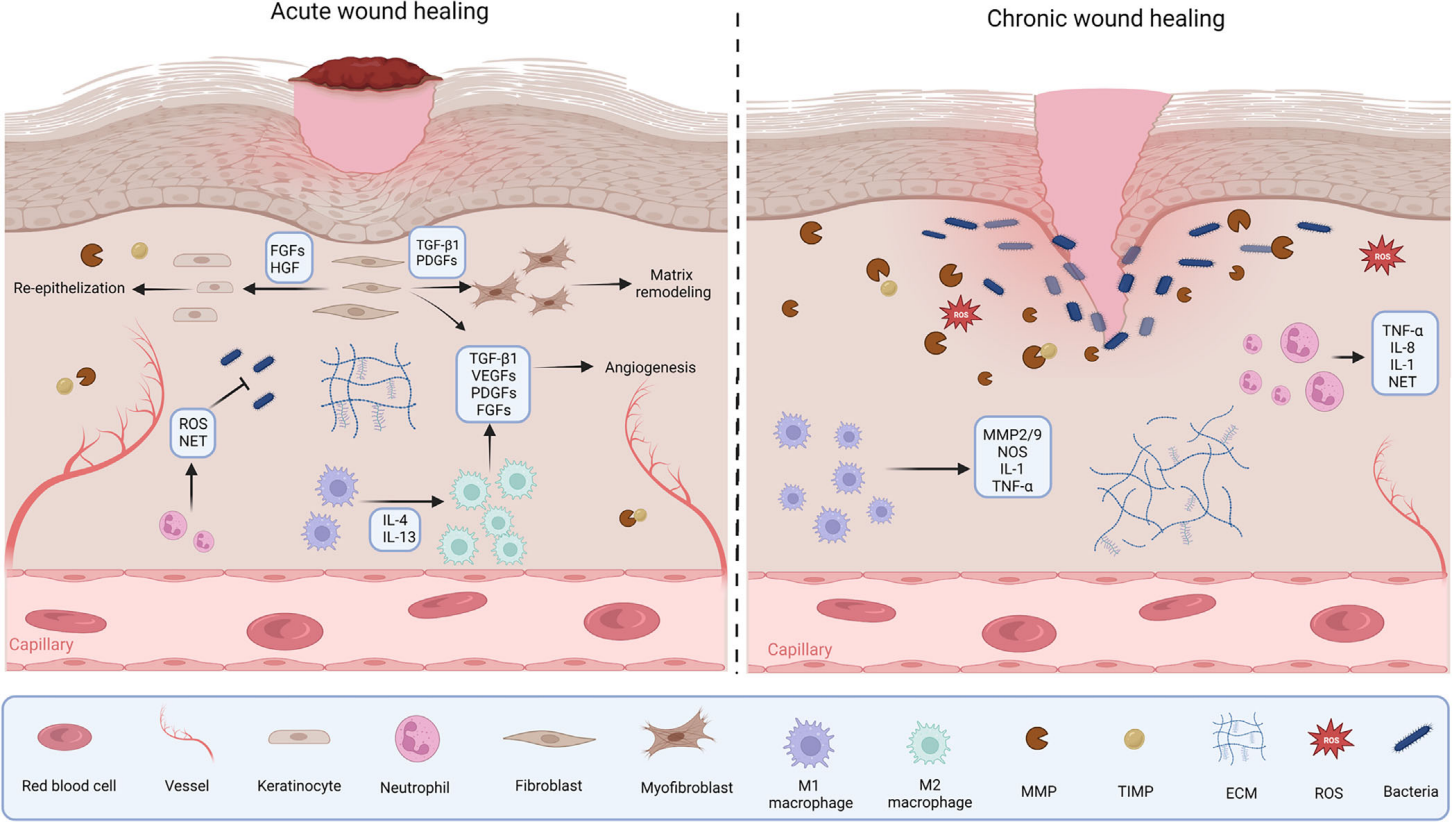
Cytokine and growth factor signaling in acute and chronic wound healing. Figure prepared using Biorender.com.

Normal wound healing relies on cooperation between biochemical mediators and inflammatory cells, and any disruption or delay in the organized progression of normal wound healing may lead to the development of a chronic wound (Figure 1). In general, a wound is classified as “chronic” if it remains open for longer than 12 weeks.^49^

Prolonged inflammation originating from repeated trauma or pressure on a wound site along with neuropathy, impaired circulation, bacterial infection, and a compromised immune response are among major factors hindering the healing of chronic wounds. ECM fragments, bacterial biofilms, and platelet‐derived factors such as TGF‐β recruit many inflammatory cells to the wound bed. The increased number of inflammatory cells in the wound bed, along with the sustained presence of TNF‐α and IL‐1β released from recruited inflammatory cells, cause increased protease production in the wound bed. Although the activity of proteases is controlled in acute wound healing processes by their inhibitors, excessive protease levels in chronic wounds surpass inhibitor levels resulting in the degradation of ECM, GFs, and receptors. Degradation of ECM by proteases not only stops the progression of the wound into the proliferative phase, but also amplifies inflammatory responses by recruiting more inflammatory cells to the wound bed.^46^, ^50^

The accumulation of inflammatory cells and subsequent accumulation of ROS adversely affect protein structure in the ECM and lead to premature cellular aging, which in turn impairs the ability of cells to function optimally and further contributes to impaired wound closure and dysregulated tissue regeneration.^51^ With the help of inflammatory cytokines, ROS trigger chain reactions in which proteases such as MMPs are increasingly activated. Nearly 30‐fold higher MMP levels have been reported in chronic wounds in comparison with acute wounds.^52^ MMPs completely degrade a majority of matrix proteins, including elastin, fibronectin, proteoglycans, and collagen, and they also degrade GFs essential for normal wound healing.^46^, ^53^ Furthermore, proteolytic degradation of tissue inhibitors of matrix metalloproteinases (TIMPs) disrupts the balance between MMPs and TIMPs, which is required for normal wound healing.^46^

Other significant factors contributing to the delayed healing process are the abnormal phenotypes of the dermal and epidermal cells that develop in chronic wounds. Lower mitogenic ability and lower numbers of GF receptors are among the abnormalities that can hinder cells from responding properly to repair signals.^46^ Macrophages in chronic wounds produce high levels of inflammatory mediators, leading to an imbalance between classically activated (M1) and alternatively activated (M2) macrophages, with M1 macrophages dominating.^54^ Similarly, irregular cytokine secretion patterns, reduced proliferative capacity, and an early shift to senescence have been reported in fibroblasts found in chronic wounds.^55^, ^56^

### 2.3 Impaired GF and cytokine signaling in chronic wounds

GFs play a crucial role in all phases of wound healing. When a GF binds to its receptor, it triggers a cascade of intracellular signaling pathways regulating cellular function. Chronic wounds are characterized by reduced concentrations of GFs, which can be attributed to increased GF degradation and cellular phenotypic changes that result in low GF expression.^20^

During the inflammatory phase, TGF‐β is one of the GFs responsible for the recruitment of inflammatory cells. Once TGF‐β1 binds to the TGF‐β receptor ІІ (TGF‐βRІІ), a chain of reactions is triggered, leading to the formation of a heteromeric receptor complex. As a result of TGF‐βRІІ activation, the downstream signaling molecules Smad2 and Smad3 initiate the formation of a complex with Smad4. This complex translocates into the nucleus, regulating genes associated with inflammation.^57^ Suppression of TGF‐β receptors and insufficient amounts of Smad2 in chronic wounds lead to disruption in the TGF‐β signaling pathway.^58^

IL‐6 is among the ILs that play a crucial role in the proliferation phase. IL‐6 is produced by macrophages, keratinocytes, fibroblasts, and endothelial cells. Following its binding to IL‐6 receptor α, IL‐6 activates the STAT–JAK signaling pathway. This activation subsequently induces VEGF and IL‐1 secretion, collagen production, macrophage and neutrophil recruitment, TGF‐β1‐induced proliferation of endothelial cells, and angiogenesis.^59^ To this end, higher levels of IL‐6 in chronic wounds as compared to acute wounds is a major contributing factor in persistent inflammation.^60^, ^61^

The expression of hepatocyte GF (HGF) and its receptor, c‐Met (mesenchymal epithelial transition factor), were found to be elevated in skin wounds. IL‐1, IL‐6, and TNF‐α activate the transcription of HGF, which in turn controls neoangiogenesis and granulation tissue formation.^61^, ^62^ Additionally, HGF inhibits inflammation resulting from VEGF activity through induction of ICAM‐1 and VCAM‐1 expression.^63^, ^64^ The development of the chronic wound phenotype depends on imbalances in activation and deactivation of the HGF/c‐Met pathway. In particular, inflammatory peptidases, such as neutrophil elastase, are responsible for HGF processing, which reduces c‐Met activation through degradation of active HGF and decreases reepithelialization and keratinocyte proliferation.^61^, ^65^ As a result, topical administration of HGF could potentially serve as a promising therapeutic option for chronic wounds.

The presence of TGF‐α, EGF, and heparin‐binding EGF, secreted by keratinocytes and macrophages, can significantly impact the process of wound healing through epithelialization. The GFs activate EGF receptors (EGFRs) on the surface of keratinocytes, triggering a series of biological responses that promote migration and proliferation of keratinocytes and lead restoration of the epithelial layer.^61^, ^66^, ^67^ EGFR signaling relies on the activity of two transcription factors, AP1 and STAT3. These factors are essential for the proper functioning of wound healing, and a deficiency of STAT3 in keratinocytes can decrease migratory and skin remodeling behaviors.^61^, ^68^ Recent studies conducted by Brem et al.^69^ revealed that keratinocytes in chronic wounds have lower levels of EGFR on cell surfaces. These authors concluded that decreased levels of EGFR on cell surfaces, and the resulting inability to properly respond to EGFR signaling, may hinder the normal process of wound healing, ultimately leading to chronic wound formation.

## 3 EXOGENOUS GF DELIVERY

As the understanding of chronic wound physiology has improved, GFs have emerged as a clear target for new therapies.^19^ Preclinical wound‐treatment studies with recombinant GFs began in the 1990s, and the field has continually progressed over the past three decades, evolving from topical application strategies to biomaterial‐based, controlled release approaches involving delivery of multiple GFs.^22^ During this time period, a few treatments attained clinical approval in the United States and elsewhere, but the majority have either failed to demonstrate efficacy in human trials or failed to reach clinical trials altogether.^70^ The field of exogenous GF delivery has been extensively reviewed multiple times over the course of its development.^20^, ^22^, ^26^, ^70^, ^71^, ^72^ Therefore, this section will focus on the most recent strategies for controlled delivery of multiple GFs, the limitations of exogenous delivery, and how these studies can inform future GF therapeutics.

### 3.1 Exogenous GF delivery in the clinic

Attaining clinical approval for chronic wound therapies has been notoriously challenging and even approved therapies lack widespread clinical use.^19^, ^73^, ^74^ In most cases, preclinical wound healing outcomes are promising, but limitations in the available animal models, the narrow scope of early stage clinical trials, and the potential shortcomings of therapies targeting single GFs contribute to the lack of clinical translation.^75^ The only FDA approved recombinant GF therapy for chronic wounds is Regranex (becaplermin gel), a topically applied recombinant PDGF treatment. However, post‐approval clinical evaluations have identified shortcomings in efficacy (e.g., reduced efficacy in infected wounds) and safety (e.g., increased cancer risks) that were not observed in tightly controlled clinical trials.^73^, ^76^, ^77^, ^78^, ^79^ Specifically, Regranex was tested and approved in neuropathic ulcers that lack infection or other complications, but neuropathic ulcers of this nature are only a small portion of chronic wounds.^75^ Additionally, long‐term repeated applications of high‐dose treatments led to an elevated risk for cancer.^80^ Other recombinant GF therapies (FGF and EGF) have been approved for use in countries outside the United States. Recombinant FGF (Fiblast Spray) is commercially available in Japan, and multiple recombinant EGF treatments (Heberprot‐P, Regen‐D 150, and Easy‐ef) are available in various other countries.^81^, ^82^, ^83^, ^84^, ^85^ However, similar to Regranex, healing outcomes from these recombinant GF therapies have been inconsistent; for example, Fiblast was not provided FDA approval because it failed to demonstrate efficacy over a placebo spray in Phase III clinical trials.^20^, ^86^, ^87^, ^88^

Due to the short half‐life of proteins in the protease‐rich wound environment, recombinant GF therapy requires repeated supraphysiological doses to produce positive effects on wound healing.^70^ However, these large doses can lead to an uncontrolled cellular response, which results in many of the side effects observed clinically, including increased risk of certain cancers.^79^, ^80^ Additionally, the heterogeneity of chronic wounds makes developing appropriate treatment regimens challenging, contributing to the variability in healing outcomes reported in the literature.^75^ While recombinant GF therapies have improved chronic wound healing outcomes in some cases, the large number of clinical failures for recombinant GFs has motivated research that addresses challenges with protein stability and temporal control of delivery, with many groups using biomaterials to improve the stability, increase the half‐life, and tune the release of GFs in the wound environment to better match the environment of acutely healing wounds.

### 3.2 Biomaterials strategies for controlled release of exogenous GFs

Delivering GFs from biomaterials offers a variety of advantages. First, the amount and timing of a cargo released to the wound environment can be more easily controlled. In addition, biomaterials offer spatial control of GF release that is not possible with topically applied GFs. Lastly, the material can protect the GFs from degradation, improve their bioavailability in the wound, and decrease the required doses, reducing side effects.^71^ The commonly tested GFs include PDGF, VEGF, EGF, and FGF, with fewer studies on others such as KGF and IGF.^20^, ^22^ Research on these particular GFs is motivated by their key roles in the wound healing cascade, as described above, and a summary of select studies presented in this section are provided in Table 1.

**TABLE 1:** Summary of exogenous growth factor therapies.

Delivery strategy	GF Cargo	Material formulation
Bulk release of directly loaded GFs	EGF	Collagen and HA sponge^89^, ^90^
		*N*‐carboxymethyl chitosan‐ and alginate‐based hydrogels^91^
		Chitosan films^92^
		Polyurethane foam^93^
	FGF	Chitosan‐crosslinked collagen sponge^94^
		Gelatin sheets^95^
		Silver crosslinked chitosan hydrogels^96^
		*N*‐isopropyl acrylamide and acrylic acid hydrogel/polyurethane composites^97^
	KGF	Chitosan‐silica hybrid dressings^98^
Nano‐ and micro‐structures in bulk materials	PDGF	Chitosan nanoparticles encapsulated in polycaprolactone electrospun fibers^99^
	VEGF	Fibrin nanoparticles in chitosan‐HA sponges^100^
	EGF	PLA nanoparticles in PLA‐based hydrogels^101^
	FGF	Poly(ethylene glycol)‐poly(dl‐lactide) electrospun core‐sheath nanofibrous mats^102^
Modified GFs in bulk materials	FGF	Chitin binding domain‐FGF in chitin films^103^
	EGF or FGF	Structurally stabilized GFs in hyaluronate/collagen dressings^104^, ^105^
Multi‐cargo approaches	VEGF and FGF	Heparin functionalized, polyethylene glycol crosslinked chitosan scaffold^106^
		PLGA nanoparticles in a fibrin‐based scaffold^107^
	VEGF and EGF	Chitosan microparticles in a dextran hydrogel^108^
	VEGF and PDGF	PLGA scaffolds containing microspheres of the same polymer^109^
		Electrospun chitosan and poly(ethylene oxide) (PEO) nanofibers containing PLGA nanoparticles^110^
	VEGF, FGF PDGF, and EGF	Gelatin nanoparticles in electrospun collagen and HA nanofibers^111^
	GFs and other cargo	Methylcellulose and chitosan hydrogel delivering VEGF, silver, and curcumin^112^
		Nanofibrous mat composed of polycaprolactone (PCL), chitosan, PEO, and collagen to delivery EGF, FGF, and silver sulfadiazine^113^
		Conditioned media from bone marrow‐derived stem cells^114^

**FIGURE 2:**
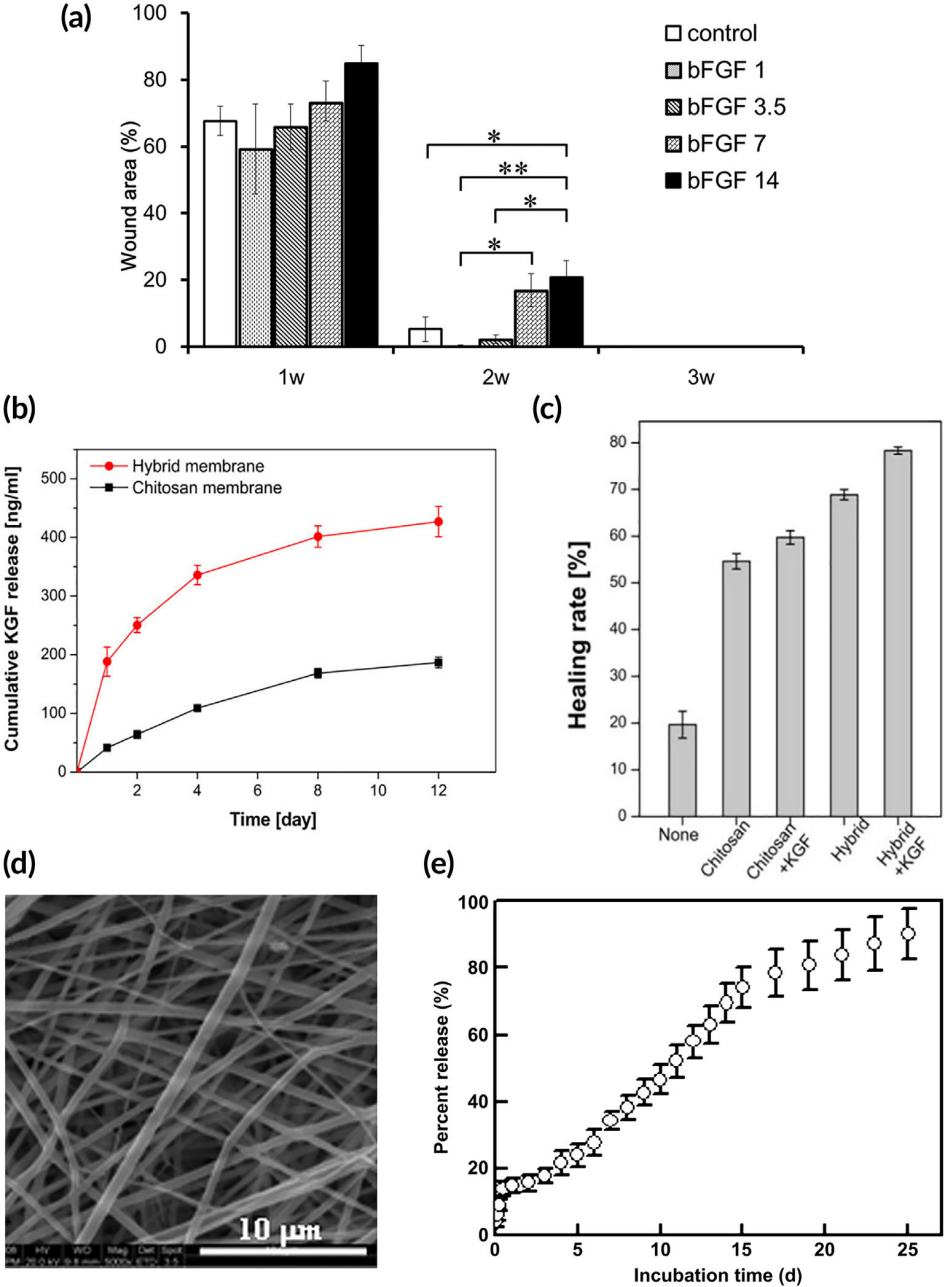
Biomaterials for single growth factor (GF) delivery. (a) Time dependent wound closure data for gelatin gel sheets loaded with varying concentrations of fibroblast GF (FGF). Adapted with permission, copyright 2016 Elsevier.^95^ (b) Release profiles and (c) healing rates for KGF loaded chitosan and silica‐chitosan hybrid wound dressings. Adapted with permission, copyright 2019 Elsevier.^98^ (d) An SEM image and (e) the associated release curve of FGF loaded poly(ethylene glycol)‐poly(dl‐lactide) electrospun fibers. Adapted with permission, copyright 2011 Elsevier.^102^

In the clinically approved Regranex formulation for topical recombinant PDGF, the GF is encapsulated in a carboxymethylcellulose hydrogel, providing some control of GF release.^20^ However, in clinical practice, repeated applications of Regranex are required for wound healing benefits, suggesting that sustained release formulations could reduce the need for multiple doses and better regulate the concentration of GF present in the wound.^70^, ^79^ Improved bulk‐release materials, which are 3D structures in the form of rigid scaffolds or less rigid, injectable hydrogels composed of bioderived or biocompatible materials, have shown preclinical promise for controlling release and treating chronic wounds. ECM materials such as gelatin, collagen, and hyaluronic acid are regularly used due to their natural biocompatibility, and these ECM materials have been shown to promote wound healing when formulated with a single GF cargo.^16^ One study demonstrated faster wound healing and increased reepithelialization and angiogenesis in a rat wound model with EGF delivery from a collagen and hyaluronic acid sponge.^89^ A later study confirmed these findings in diabetic mouse models, using a similar collagen/HA sponge with EGF, but with the addition of a vitamin C derivative.^90^ Other studies delivering FGF from various ECM‐derived 3D structures have demonstrated that materials‐based, bulk‐release methods for GF delivery improve wound healing outcomes over administration of GF solutions or wound dressings alone (Figure 2a).^94^, ^95^ Bioderived materials such as chitosan and alginate (Figure 2b,c) have also been widely explored in wound models as options for encapsulating and releasing GFs, and several studies have demonstrated more rapid and complete reepithelialization and increased granulation tissue formation after application of these GF‐loaded dressings as compared to application of the dressing or GFs alone.^91^, ^96^, ^98^ However, one study using excisional wounds in porcine models was unable to show significant differences in wound healing following application of EGF‐delivering chitosan films versus unloaded chitosan films.^92^

In addition to bioderived materials, biocompatible synthetic polymers have also been used to deliver exogenous GFs, and polyurethane has been successfully used to deliver EGF to diabetic wound models in rats.^93^ Polyurethane also was used in a composite hydrogel to deliver FGF to murine skin wounds, and this treatment resulted in a reduction in scarring and faster wound healing than no treatment, hydrogel only, and GF‐only controls.^97^ However, by physically encapsulating the GFs, control over release is limited to tuning the diffusion properties and degradation of the surrounding material, resulting in the burst release profiles reported in many of these material formulations.

To improve control over GF release profiles, various strategies have incorporated micro‐ or nano‐scale particles or other nanostructures into wound dressings. For example, using electrospun fibers containing PDGF‐loaded chitosan particles, Piran et al. showed sustained release of PDGF and resulting fibroblast migration in vitro.^99^ Another in vitro study demonstrated steady release of VEGF from fibrin nanoparticles encapsulated in chitosan‐HA sponges for 48 h, leading to enhanced tube formation, a marker of angiogenesis.^100^ Li et al. employed polylactic acid (PLA) to form nanoparticles encapsulating EGF and curcumin, which were then dispersed in a PLA‐based hydrogel, reducing the burst release of EGF over the first 2 days (100% release of free EGF compared to 35% of encapsulated EGF) and accelerating wound closure and granulation in rat models.^101^ Another method for controlling GF release has employed nanostructured materials, in which GFs are incorporated into different layers of the material, providing tunable release. In one example, Yang and coworkers developed electrospun mats consisting of nanofibers with a core‐sheath structure (Figure 2d). When loaded with FGF, this nanostructure sustained release for 25 days and minimized burst release in the first 24 h (Figure 2e). In diabetic rat models, the FGF‐loaded mats resulted in more rapid and more complete healing.^102^ Moreover, protecting GFs from the protease‐rich chronic wound environment provided the benefits of extended half‐life and prolonged activity.

While many particle‐scaffold systems offer some degree of protection for GFs, modifying the GFs themselves can further extend their lifetime in the wound. Wang et al. used FGF modified with a chitin‐binding domain to increase FGF retention in chitin films, leading to slower release and improved vascularization when the modified chitin films were implanted subcutaneously in a rat model.^103^ Choi et al. stabilized the structure of EGF and FGF by introducing disulfide bonds and increasing the hydrophobicity of the proteins before loading them into a hyaluronate/collagen matrix. The modified proteins were more stable than commercially available versions of the same GFs and exhibited similar activity. These matrices sustained GF release for 21 days and were shown to improve would closure, reepithelialization, and collagen deposition over untreated and matrix only controls.^104^, ^105^

Strategies focused on delivery of a single cargo have continually advanced, particularly for recombinant GF delivery. Compared to commercially available products, current preclinical therapies provide controlled temporal release, improved spatial release, and increased GF availability in the wound for up to 3 weeks or more. However, because single GF therapies cannot replicate the GF‐rich environment required for wound healing, many studies have explored the delivery of multiple GFs to improve wound healing outcomes.

### 3.3 Benefits of multi‐cargo delivery

**FIGURE 3:**
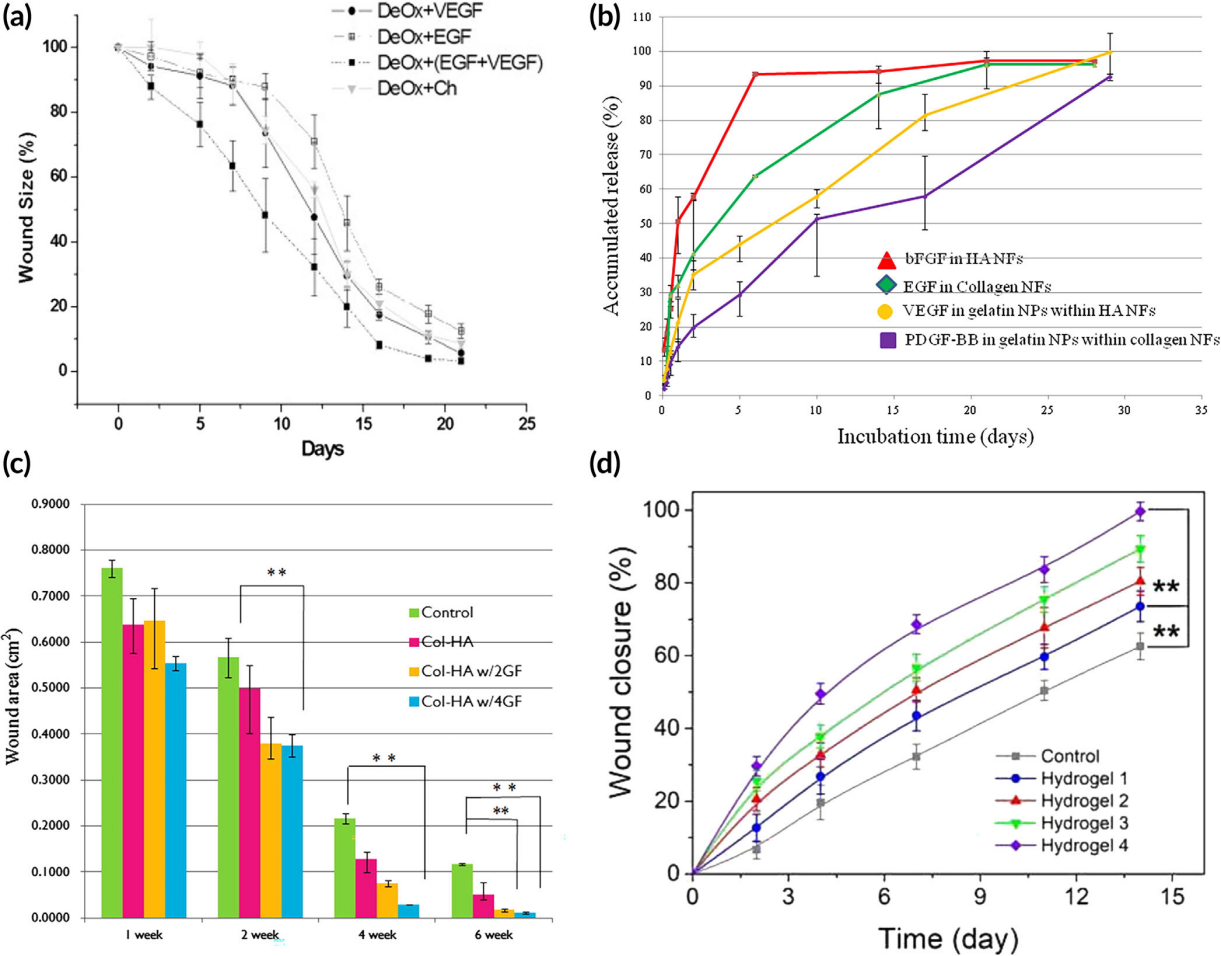
Exogenous multi‐growth factor (GF) delivery approaches. (a) Faster wound closure is obtained with a combination of VEGF and EGF using a system with chitosan microparticles loaded into dextran hydrogels. Adapted with permission, copyright 2013 Elsevier.^108^ (b) Release curves for GFs encapsulated in collagen and HA nanofibers (NFs) or gelatin NPs within the collagen and HA NFs and (c) the associated wound closure data. Adapted with permission, copyright 2014 Elsevier.^111^ (d) Wound closure data for methylcellulose‐chitosan hydrogels loaded with no cargo (Hydrogel 1), silver and curcumin loaded polydopamine (PDA) NPs (Hydrogel 2), VEGF (Hydrogel 3), and VEGF with silver and curcumin loaded PDA NPs (Hydrogel 4). Adapted with permission, copyright 2023 Elsevier.^112^

The controlled delivery of multiple GFs to the wound is a promising approach for improving wound healing therapies. Most multi‐GF delivery approaches deliver both GFs simultaneously, relying on diffusion and material degradation for control. While this allows for the GFs to act synergistically and has been shown to improve healing outcomes over single GFs, it does not accurately mimic the typical wound healing cascade. Like single GF delivery strategies, a variety of materials have been used to encapsulate and release multiple GFs. Vijayan et al. used a chitosan scaffold crosslinked with polyethylene glycol and functionalized with heparin to bind and release VEGF and FGF. Both GFs had similar release profiles in vitro and improved wound healing over an unloaded control in vivo.^106^ VEGF and FGF were also used in a fibrin‐based scaffold containing poly(lactic‐*co*‐glycolic) acid (PLGA) nanoparticles. However, this work demonstrated that the GF‐loaded nanoparticles did not perform significantly better than bulk encapsulation of the GFs within the scaffold, although the GF‐loaded scaffolds performed better than the unloaded scaffolds.^107^ Co‐delivery of VEGF and EGF from chitosan microparticles loaded in dextran hydrogels demonstrated more rapid wound closure compared to single GF delivery and bolus treatments, indicating synergy between VEGF and EGF (Figure 3a).^108^

Building upon this work, other research has explored strategies for controlling the individual release of distinct GFs. In 2001, the Mooney group developed a polymeric material for GF delivery with two release profiles, which they used to deliver VEGF and PDGF, resulting in improved vascularization over individual GFs and bolus delivery.^109^ Following this work, Xie et al. used electrospun nanofibers containing VEGF and PLGA nanoparticles loaded with PDGF to yield burst release of VEGF in the first 24 h and sustained release of PDGF over the course of a week, quickening the closure of full thickness skin wounds on Sprague–Dawley rats.^110^ Lai et al. used a multi‐functional material based on electrospun fibers composed of collagen or HA and gelatin nanoparticles to load and release PDGF, VEGF, EGF, and FGF. EGF and FGF were loaded directly into the collagen and HA nanofibers, respectively, and VEGF and PDGF were encapsulated in gelatin nanoparticles and then loaded into HA and collagen nanofibers, respectively. Each GF exhibited a different release profile: FGF was released most rapidly with over 90% cumulative release in 5 days; EGF showed 90% release between 15 and 20 days; and VEGF and PDGF achieved 90% release after 20 and 25 days, respectively (Figure 3b). Compared to the controls, GF‐loaded scaffolds increased the rate of wound closure and angiogenesis, with a formulation comprising four GFs enabling more rapid healing than a combination of only FGF and EGF (Figure 3c).^111^

Furthermore, GFs have been incorporated with other cargo to provide multifunctional wound dressings. Adding antimicrobials to GF formulations, such as silver and its derivatives,^96^, ^112^, ^113^ or anti‐inflammatory agents, such as curcumin,^101^, ^112^ can prevent infection and reduce inflammation associated with nonhealing wounds. In one example, a pH‐responsive methylcellulose and chitosan hydrogel was used to deliver VEGF, curcumin, and silver to increase angiogenesis, reduce inflammation, and prevent infection (respectively), leading to nearly 100% wound closure in 14 days compared to only 75% wound closure for unloaded hydrogels (Figure 3d).^112^ Alternatively, cell‐based therapies gained traction for their ability to supply an array GFs with a single application. Apligraf and Dermagraft remain the only FDA approved cell‐based therapies, but others have been tested and approved elsewhere in the world, with fibroblasts and keratinocytes as the primary choice in these interventions.^115^ Notably, Holoderm and Kaloderm, both keratinocyte‐based products, are used to treat DFUs in Korea.^116^

Moreover, the advantages of stem cells in regenerative medicine have motivated researchers to pursue their potential as a method for supplying GFs to the wound, and preclinical exploration of these approaches has been discussed extensively by Kucharzewsk et al.^117^ While a bolus injection of stem cells has improved wound healing outcomes in some preclinical studies, most attempts have failed because of low cell survival rate in wound bed, lack of cell‐ECM attachment, and additional tissue damage, leading researchers to focus on encapsulating stem cells in an ECM‐derived matrix.^118^, ^119^ One study demonstrated accelerated wound closure in an irradiated mouse model with human adipose‐derived stem cells co‐cultured with endothelial cells on a collagen peptide bioscaffold.^120^ In addition to using stem cells to promote wound healing, the use of the stem cell secretome (such as exosomes or conditioned media), has also been employed as a promising therapeutic approach in various studies. One study demonstrated accelerated wound closure and angiogenesis in a diabetic mouse model treated with exosomes derived from induced pluripotent stem cells, and fibroblasts isolated from these mice showed increased migration and proliferation in vitro.^121^ In another study, Chen et al. concluded that conditioned medium collected from bone marrow‐derived mesenchymal stem cells grown in hypoxic conditions can significantly accelerate wound healing in Balb/c nude mice.^114^

**FIGURE 4:**
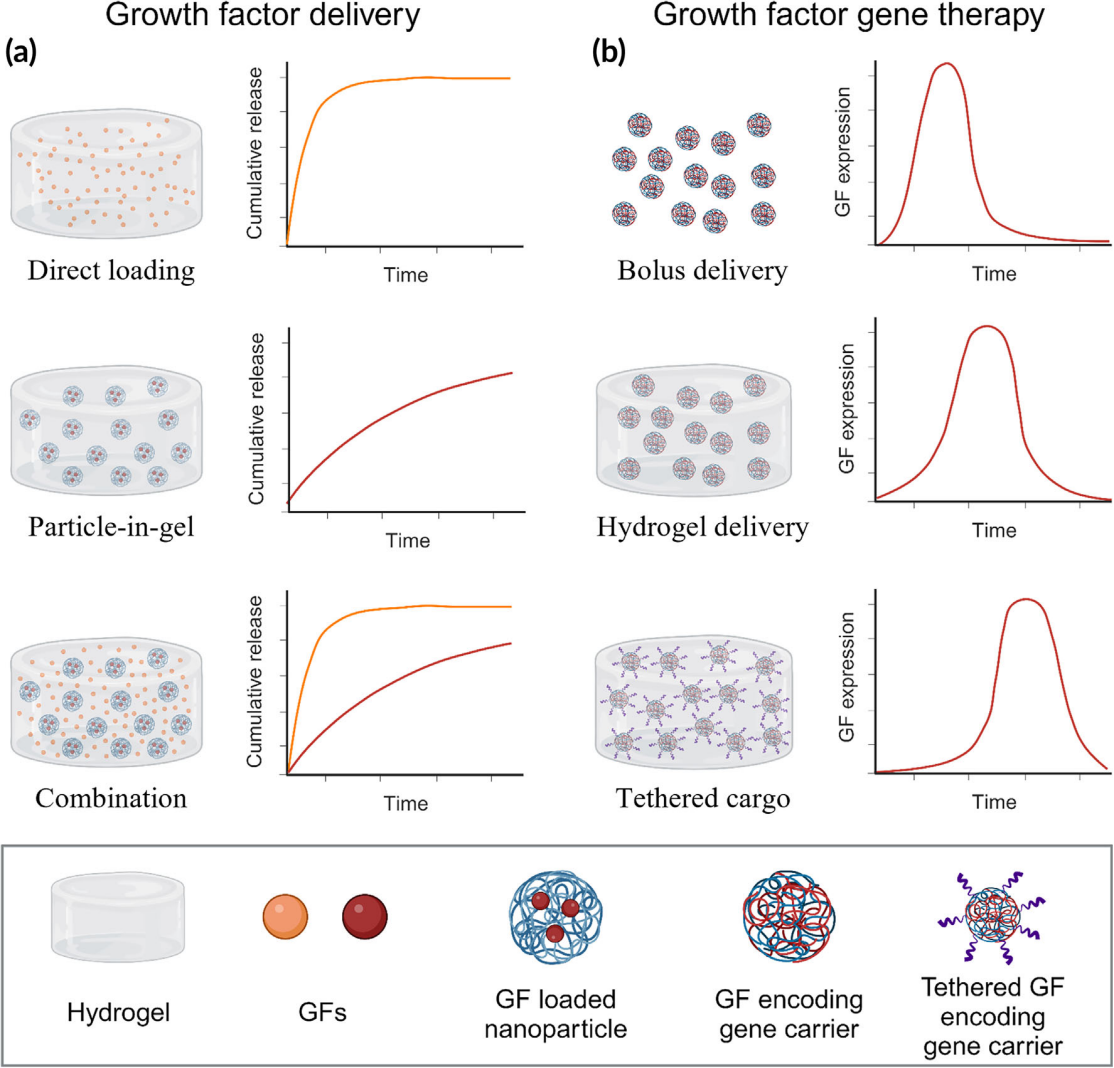
Commonly observed release and growth factor (GF) expression profiles from various delivery approaches. (a) Exogenous growth factor delivery approaches including direct loading, particle‐in‐gel loading, and combination systems. (b) Gene therapy approaches including bolus delivery, hydrogel delivery, and tethered gene carrier systems. Figure prepared using Biorender.com.

While delivering multiple cargos has been effective for improving wound healing outcomes in preclinical studies, a few key challenges have prevented clinical translation. Despite many efforts to increase stability and protect GFs in the wound environment, the protease‐rich environment still prevents long‐term GF availability, resulting in the need for multiple high doses of GF for measurable therapeutic effect and raising concerns about side effects and safety. Additionally, determining the timing of gene expression remains a challenge in these systems, and there are limited studies comparing wound healing outcomes across different release profiles. For example, one study delivered VEGF with nearly complete release in 24 h,^110^ but another study delivered VEGF gradually, reaching 90% release at 20 days.^111^ While various different release profiles, schematically represented in Figure 4a, have been shown to improve wound healing outcomes in animal models, it is difficult to compare results across studies, and more empirical results are needed to determine the appropriate temporal profiles for GF‐ therapies. Overall, research on exogenous GF delivery has demonstrated clear benefits for at least partially correcting impaired healing, providing a framework that can be used to build the next generation of chronic wound therapeutics.

## 4 GF GENE DELIVERY

The challenges facing recombinant GF delivery, including protein stability, off‐target effects, and short half‐life in the wound environment, have motivated the pursuit of GF gene delivery strategies (Table 2) as an alternative. As discussed above, improved stability is the focus of many efforts in the exogenous GF delivery space.^71^ In this context, gene delivery offers potential advantages over protein delivery by enabling cells to endogenously produce GFs, thereby providing a continuous source of fresh GF.^70^ Moreover, gene carriers can be targeted to specific cells, which improves spatial control of GF delivery, increases therapeutic efficacy, and reduces off‐target effects compared to exogenous GF delivery.^132^ Furthermore, sustained GF signaling is required to drive healing in chronic wounds, but exogenous GFs can only provide signaling for as long as the dose is present in the wound environment.^57^ In contrast, gene therapy can provide tunable GF expression profiles, as schematically illustrated in Figure 4b, without the need for supraphysiological doses. The advantages of GF gene delivery have motivated studies of its potential as a new class of wound‐healing therapeutic, but challenges with efficient transfection and regulatory concerns have prevented clinical approval of any gene‐delivery‐based wound healing approaches to date.^25^

**TABLE 2:** Summary of growth factor and cytokine gene therapies.

Delivery strategy	Gene Cargo	Material formulation
Bolus delivery of gene carriers	Sonic hedgehog	PBAE polyplexes^122^
	VEGF mRNA	Lipid nanopartilces^123^
	TNF‐α siRNA	Lipidoid nanoparticles^124^
Gene carriers in hydrogels	VEGF	Dextran, HA, and β‐cyclodextrin hydrogels loaded with PEI polyplexes^125^
	Reporter mRNAs	PBAE polyplexes in PBAE‐poly(ethylene glycol) hydrogels^126^
	Reporter siRNA	Porous polyurethane scaffolds loaded with methacrylate‐based deblock copolymer nanoparticles^127^
Modified gene carriers in hydrogels	PDGF	Collagen‐fibrin hydrogels containing CMP modified PEI polyplexes^128^
	VEGF	Collagen‐HA hydrogels containing CMP modified PEI polyplexes^129^
Multigene delivery	VEGF and angiopoitin‐1	Scaffolds made from PLGA mesh reinforced with collagen and chitosan loaded with PEI polyplexes^130^
	VEGF and PDGF	PLGA nanospheres^131^

### 4.1 Viral gene delivery strategies

Viral vectors have been extensively explored as delivery vehicles for genetic cargo, but their use in wound healing has been relatively limited. Compared to other gene delivery strategies, viral vectors are advantageous for their high infectivity, transducing upward of 95% of cells depending on the vector type.^133^ Moreover, because of the increased number of senescent cells in the chronic wound environment, it is beneficial to have a vector that can transfect these nondividing cells.^134^ Other important properties to consider in the design of viral vectors for wound healing include immunogenicity, stability, gene insert length, and transient expression.^23^ Most studies using viral vectors have employed adenoviruses, adeno‐associated viruses (AAVs), or retroviruses, each of which offers a unique set of advantages and disadvantages.

Adenoviruses are the most frequently explored viral vector for wound healing applications.^135^ Delivering double‐stranded DNA, adenoviral vectors are favored for their high infection efficiency, transient gene expression, and ability to infect both dividing and nondividing cell types.^134^, ^136^ One application of adenoviral vectors delivered DNA encoding PDGF and demonstrated increased wound closure compared to a salt solution control in ischemic rabbit ear models.^137^ This formulation was eventually tested in human trials but has yet to be clinically approved.^138^, ^139^ Other studies using adenoviruses have demonstrated improved wound healing by VEGF gene transfer in *db*/*db* genetically diabetic mice and CD1 streptozotocin‐induced diabetic mice.^140^, ^141^ However, there are inconsistent findings using similar approaches, including one study involving porcine models that demonstrated high expression of VEGF from adenoviral transduction but no subsequent improvement in wound closure.^142^

AAVs are another commonly used viral vector that contain single‐stranded DNA as their cargo. As opposed to adenoviruses, AAVs are much less immunogenic, increasing their safety while maintaining high infectivity, transient gene expression, and the ability to infect dividing and nondividing cells. The advantages of AAVs have led to success in preclinical studies using VEGF. A pair of studies, one using an acute wound model in Wistar rats and the other using a diabetic model in *db*/*db* mice, demonstrated large increases in vascularization and improved wound closure,^143^, ^144^ but despite promising preclinical results, limitations in the size of the encoded gene and yields from recombinant viral vector production have slowed the progress of adenoviral vectors toward clinical relevance.^132^, ^135^

Retroviruses were the first viral vectors developed. Retroviruses encode single‐stranded RNA cargo that integrates within the host genome following reverse transcription, and as such, they have primarily been explored in wound healing applications for ex vivo transduction of cells prior to introduction of the transduced cells in the wound.^23^, ^145^, ^146^ In one study, retroviruses were used to genetically modify keratinocytes to overexpress PDGF ex vivo before reintroducing them into the wound. The transduced cells improved vascularization and reepithelialization compared to controls in a murine model.^147^ Clinical translation, however, has been impeded by the low transduction efficiency of these vectors and safety concerns about mutagenesis from host‐genome integration.^132^

In addition to safety concerns, viral gene therapies have also been limited by difficulties in tuning GF gene expression outcomes in alignment with desired GF concentration profiles in the wound environment.^25^, ^135^, ^148^ Additionally, existing viral GF gene therapy approaches do not consistently result in improved wound healing, potentially because delivery of a single GF gene therapy is insufficient for addressing the complex needs of the chronic wound bed; however, to our knowledge, polycistronic viral vectors (i.e., viral vectors encoding multiple genes) have only been used in one study to date where both VEGF and FGF plasmids were delivered in a single AAV to skin wounds on genetically diabetic mice, resulting in more rapid wound healing than delivery of AAVs that contained only a single GF plasmid.^149^ While viral vectors have considerable potential in other therapeutic spaces, they have not yet achieved clinical relevance in chronic wound healing applications.

### 4.2 Nonviral gene delivery strategies

Nonviral vectors offer opportunities to overcome the challenges with viral gene delivery due to their chemical versatility and ability to be delivered on or within biomaterial scaffolds routinely used in wound management. Chemical transfection methods are most common for wound healing approaches and generally involve the construction of nanoparticles formed when polycationic materials, including lipids or polymers, interact electrostatically with the anionic phosphodiester backbone of DNA to form a lipoplex or polyplex, respectively. In addition, encapsulation of polyplexes or lipoplexes within a biodegradable hydrogel yield hydrogel/scaffold formulations that can both provide support for cellular growth and also deliver genes to cells nearby.^150^

#### 4.2.1 Polymeric nanoparticles

**FIGURE 5:**
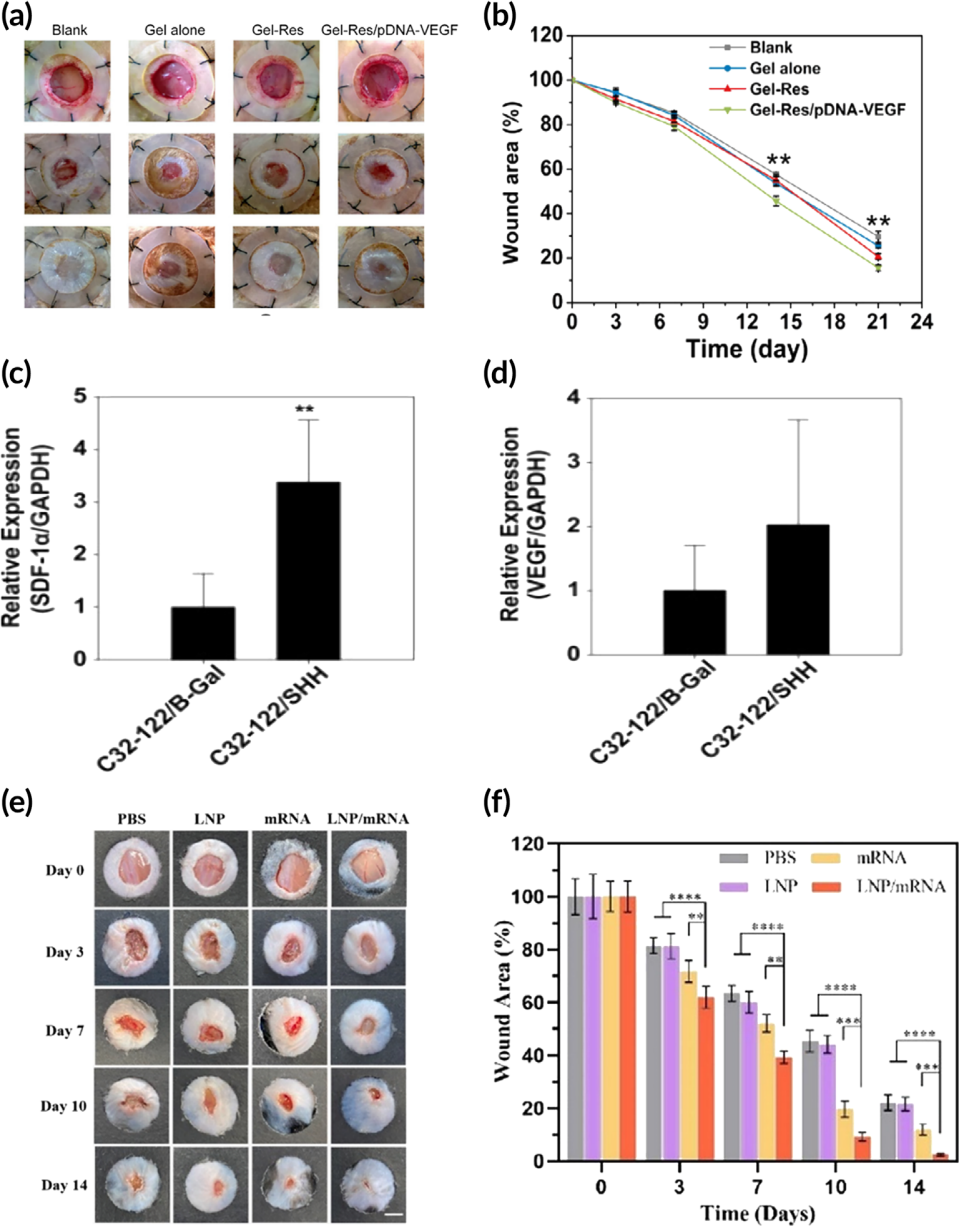
Examples of various polymer and lipid‐based gene delivery strategies. (a) Wound closure images and (b) data for methacrylic acid anhydride modified hyaluronic acid/*N*‐hydroxymethylacrylamide modified dextran hydrogels encapsulating no cargo (Gel alone), resveratrol (Gel‐Res), or resveratrol and PEI/plasmid DNA encoding VEGF (Gel‐Res/pDNA‐VEGF). Adapted with permission, copyright 2019 Elsevier.^125^ Real‐time PCR analysis of (c) stromal cell‐derived factor‐1α (SDF‐1α) and (d) VEGF for PBAE polyplexes loaded with sonic hedgehog and β‐galactosidase. Adapted with permission, copyright 2012 Elsevier.^122^ (e) Wound closure images and (f) data for ionizable lipid‐based nanoparticles (LNP), VEGF‐mRNA (mRNA), and ionizable lipid‐based nanoparticles encapsulated with VEGF‐mRNA (LNP/mRNA). Adapted with permission, copyright 2023 Elsevier.^123^

Cationic polymers present a viable option for nonviral gene delivery due to their broad chemical diversity and functionality. Polyethylenimine (PEI) is among the most commonly used polymers in gene delivery due to its recognized effectiveness in transfection. The positive charge of PEI enables condensation of negatively charged genetic material and facilitates cellular uptake through electrostatic interaction with the negatively charged cell membrane.^151^, ^152^ Additionally, the ability of PEI to facilitate endosomal escape through the proton sponge effect (in which an increase in the number of protonated amines leads to endosomal swelling and rupture through an influx of negatively charged ions) makes it attractive for applications in which the therapeutic outcomes depend on payload delivery to the cytoplasm or nucleus.^152^, ^153^ One study demonstrated increased angiogenesis in a rat full‐thickness wound model treated with gene‐activated dermal scaffolds loaded with PEI/plasmid DNAs encoding VEGF and angiopoietin‐1, compared to scaffolds loaded with plasmid VEGF alone or with the plasmid VEGF/angiopoietin‐1 chimeric plasmid alone.^130^ In another study, Wang et al. concluded accelerated wound healing, as evidenced by increased angiogenesis and decreased inflammation, in a splinted excisional burn wound model,  when the wound was treated with a hydrogel scaffold loaded with resveratrol and PEI/plasmid DNA encoding VEGF (Figure 5a,b).^125^ Despite these promising features, the high cationic charge density of PEI can result in cytotoxicity that originates from cell membrane disruption through electrostatic interactions.^152^, ^154^, ^155^ As a result, while the high charge density of PEI facilitates DNA condensation and transfection, its potential impact on cell viability must be carefully considered and balanced in order to optimize its application in gene therapy. In efforts to address this issue, hydrophobic moieties such as cholesterol, lipids, and steric acid have been incorporated into PEI‐based formulations to improve transfection via hydrophobic interaction between PEI‐based polyplexes and cell membranes.^156^ In efforts to address the lack of biodegradability of PEI,^157^, ^158^ various methods to incorporate stimuli‐responsiveness, such as the crosslinking of low‐molecular‐weight PEI with reducible disulfide bonds or its conjugation with ester linkages, have been proposed.^159^, ^160^

In recent years, extensive investigations have been dedicated to gene delivery platforms based on poly (β‐amino esters) (PBAEs). Unlike their nonbiodegradable counterparts, PBAEs possess a polyester backbone enriched with tertiary amine groups, which endows them with beneficial properties such as rapid degradation and enhanced water solubility.^161^, ^162^ The biodegradability of PBAEs minimizes concerns about long‐term toxicity and accumulation in vivo, thus offering a promising alternative for clinical translation. One study demonstrated increased expression of VEGF, angiogenic GF, and stromal cell‐derived factor‐1α chemokine in a full‐thickness wound model treated with PBAE/sonic hedgehog polyplexes (Figure 5c,d).^122^ A later study used PBAE polyplexes in a prototype wound dressing to control delivery to human dermal fibroblasts of both mRNA and DNA encoding for green fluorescent protein.^126^

While PBAEs alone demonstrate potential as gene delivery vectors, their rapid biodegradability has posed challenges for sustained in vivo functionality.^163^ The conjugation of PBAEs with PEI as a nonbiodegradable polymer offers an opportunity to compensate for the inherent biodegradability of PBAEs.^164^ Simultaneously, the presence of ester groups in PBAEs, which are susceptible to hydrolysis, maintain their low molecular toxicity and ensure their suitability for safe biomedical applications.^161^ Furthermore, transfection efficiency of PBAEs can be greatly influenced by the buffering system, imposing significant constraints on its widespread application. For instance, it was reported that in contrast to monovalent buffering systems, such as sodium acetate (NaAc), divalent buffering systems such as magnesium acetate and calcium acetate enhanced the transfection efficiency of PBAE‐based vectors.^165^

#### 4.2.2 Lipid particles

Lipids used for gene therapy applications include amphiphilic lipids with amine head groups or quaternary amine salts for binding to negatively charged genes and hydrophobic tails composed of cholesterol groups or alkyl chains that form a protective barrier around the core and interact with the cell membrane.^166^ In general, unsaturated alkyl or cholesterol chains are preferred for gene delivery because they improve lipid membrane fluidity and facilitate the delivery of genetic components into target cells.^167^ Their ability to function as a detergent (i.e., destabilizing the cell membrane and facilitating gene release into cytoplasm) and proton sponge have resulted in lipid‐based nanoparticles becoming a popular choice for many gene delivery systems.^168^ One study demonstrated improved epithelization, increased vessel density, and increased deposition of oriented collagen in diabetic wounds treated with ionizable lipid‐based nanoparticles encapsulated with VEGF‐mRNA (Figure 5e,f).^123^ More recently, a combinatorial library strategy has been employed to form a new class of lipid‐like materials termed “lipidoids.” Lipidoids are fabricated through conjugation of commercially available amines with epoxide, acrylamides, or lipophilic acrylates.^169^ The major advantage of lipidoids over other lipid‐based delivery materials is their straightforward and cost‐effective synthesis, which does not require harsh solvents, effectively reducing many purification and concentration requirements. Another major advantage is the structural diversity present in the libraries,^170^ which has enabled correlations between structure and function of delivery systems. These combined factors position lipidoids as an interesting polymeric delivery vehicle for fine‐tuning properties and performance. One study presented an alternative treatment approach utilizing topically administered lipidoid nanoparticles loaded with siRNA targeting TNF‐α to promote healing of DFU in an in‐vitro macrophage/fibroblast co‐culture model.^124^

While nonviral gene delivery systems hold great potential for wound healing with their intrinsic non‐immunogenic properties, they have fallen short in maintaining prolonged delivery at the site of injury, resulting in off‐target delivery and leading to potential side effects.^171^, ^172^ Furthermore, the limited gene transfection efficiency of nonviral systems has been regarded as a major impediment to their widespread application.^173^ Accordingly, approaches are still needed to enhance sustained delivery and in vivo stability in order to compensate for low transfection efficiency. In addition, approaches need to be able to accommodate the extended and multiphasic healing periods. To this end, a desirable approach would be one that can both effectively retain genetic payload until cells initiate wound repair and also transfer genes with spatiotemporal control.

#### 4.2.3 Biomaterial‐based gene delivery systems

The past decade has witnessed an increasing interest in the use of biomaterials as versatile platforms which can function as both a delivery agent and a cell‐adhesive scaffold.^174^, ^175^, ^176^ Specifically, biomaterials such as collagen, gelatin, fibrin, and hyaluronic acid have demonstrated the ability to enhance GF stability, promote cell recruitment and differentiation, and facilitate GF delivery in a sustained and controllable manner during wound healing.^128^, ^129^, ^176^, ^177^, ^178^ Harnessing the potential of these biomaterials in hydrogels holds great promise for the development of innovative wound healing strategies which can effectively promote wound repair and regeneration. One key advantage of such hydrogels is their ability to provide a protective environment for gene‐loaded nanoparticles, shielding them from protein‐rich conditions commonly found in wound beds. The release of genes from the hydrogels is mediated through matrix degradation and/or diffusion, and hydrogel encapsulation can thus facilitate the targeted delivery of entrapped genes to nearby cells, resulting in continuous and localized gene expression.^179^, ^180^ Additionally, the hydrogels can achieve healing outcomes at significantly lower doses of GFs as compared to traditional topical administration.^181^, ^182^, ^183^

**FIGURE 6:**
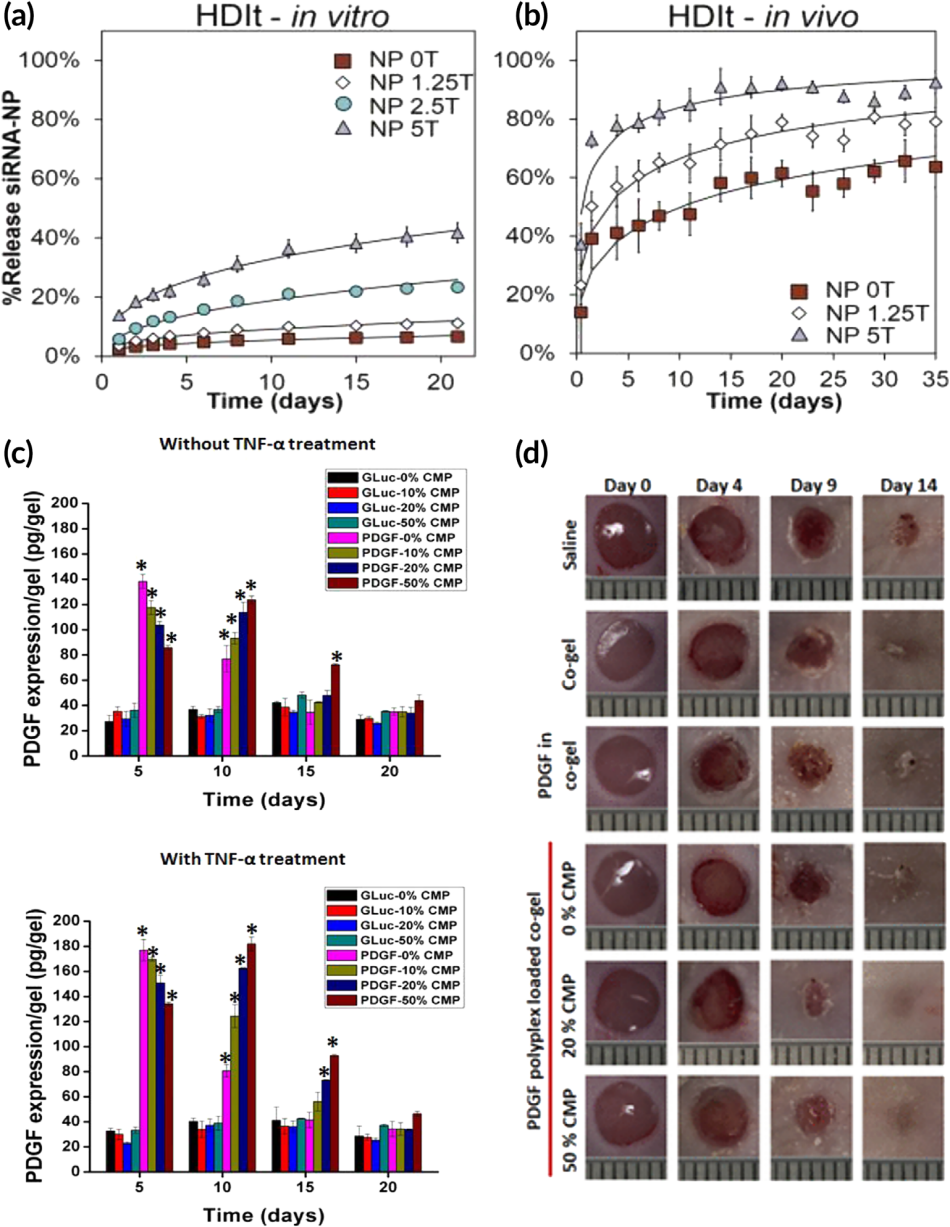
Examples of various biomaterial‐based gene delivery strategies. (a, b) Temporal release of siRNA loaded nanoparticles with varying amount of trehalose from porous polyester urethane scaffold (a) in vitro and (b) in vivo. Adapted with permission, copyright 2013 John Wiley and Sons.^127^ (c) PDGF expression of NIH 3T3 cells seeded on collagen‐fibrin co‐gels loaded with polyplexes of different CMP modifications (0, 20, and 50%) and (d) wound closure images of mice treated with saline, collagen‐fibrin co‐gels, PDGF loaded co‐gel, and PDGF polyplex loaded co‐gel. Adapted with permission, copyright 2020 American Chemical Society.^128^

Incorporation of gene‐loaded nanoparticles in hydrogels can serve as a means for influencing cellular behavior. Nelson et al. presented a temporally controlled delivery system based on porous polyester urethane scaffold loaded with siRNA‐loaded nanoparticles. Modulating the amount of trehalose added during the scaffold synthesis affords the opportunity to tune the delivery system based on the expression of therapeutically targeted genes (Figure 6a,b).^127^ Our laboratories have developed a new biomaterials approach for gene delivery in wounds by conjugating collagen‐mimetic peptides (CMPs) to polyplexes and exploiting the ability of CMPs to form triple helices with collagen as a means to sequester the polyplexes in collagen‐based matrices.^184^, ^185^, ^186^ In the absence of cells, polyplexes are stable in the collagen matrices for several weeks,^184^ whereas cellular expression of MMPs resulted in efficient release, uptake, and gene expression (Figure 6c,d).^48^, ^128^, ^187^ Furthermore, the application of CMP‐modified polyplexes loaded with plasmid DNA encoding GFs such as PDGF^128^ or VEGF^129^ improves multiple aspects of wound healing including enhanced collagen deposition, increased blood vessel formation, improved granulation tissue formation, and more rapid reepithelialization.

Hydrogels containing nanoscale structures such as nanoparticles also can show improved hydrogel stability.^188^ Although it is possible to manipulate mechanical properties of hydrogels through changes in density and chemical composition of cross‐linkers, high levels of cross‐linking can impede important cellular processes such as proliferation and migration.^189^ The incorporation of nanoparticles, including dendrimers, polyplexes, lipoplexes, and carbon‐ and ceramic‐based nanoparticles into hydrogels has enabled the development of three‐dimensional structures^128^, ^129^, ^184^, ^190^, ^191^ that can mimic some of the functions of natural tissues, making them highly suitable for tissue engineering and gene delivery applications.

Nanoparticle and hydrogel composite systems also provide the potential for controlled multigene delivery; however, this approach has not been widely explored in investigations of skin wound healing. Nearly all studies evaluating multiple GFs use the exogenous delivery methods described previously, and there are only a few published studies that report the delivery of more than one GF gene in chronic wound models. One study demonstrated increased angiogenesis, granulation thickness, and collagen deposition, along with a significantly reduced wound area, when diabetic ulcers in rat models were treated with the co‐delivery and expression of PDGF and VEGF genes from PLGA nanospheres.^131^ Outside of studies of skin wound healing, particularly in bone tissue engineering, researchers have successfully delivered multiple GF‐encoding genes. In two studies from the same group, bone morphogenetic protein 2 (BMP‐2) and VEGF plasmids were delivered to critical bone defects in rats using polymeric gene carriers in collagen‐based scaffolds, resulting in dramatically improved bone regeneration compared to the controls.^192^, ^193^ However, none of these studies provide a complete comparison between their dual gene delivery and a single gene delivery control. Additionally, the previous studies did not deliberately control the release and expression of each gene individually, leaving the challenge of distinct kinetics largely unaddressed by current work.

## 5 CONCLUSIONS

GF‐ and cell‐based therapeutics for treating chronic wounds have become a major area of exploration over the past quarter‐century. While exogenous GF delivery has produced promising preclinical outcomes, only one therapeutic has attained FDA approval. The most recent studies on exogenous GF delivery have focused on controlling the release and improving the stability of GFs. However, the large doses, short‐half lives, and potential off‐target effects still prevent the translation of exogenous GF therapeutics. Cell therapies have also shown promise in the preclinical setting, but challenges with cell survival and immune responses have slowed progress toward clinical implementation.

Many shortcomings of exogenous GF delivery and cell therapies can be addressed through gene therapy approaches. Viral vectors initially gained some traction, reaching early phase clinical trials in one case, but the lack of further exploration is likely due to a combination of safety concerns and challenges in achieving tunable expression. Nonviral gene delivery, on the other hand, offers increased tunability, making it a more attractive approach for wound healing applications. However, nonviral gene delivery approaches are challenging to optimize and limited by low transfection efficiencies. Much of the research on nonviral gene delivery is in the early stages of development and significant progress is needed to provide clinically relevant therapeutics.

As the understanding of chronic wound physiology has improved, the need for delivering multiple GFs has become clear, but controlling the expression profiles of multiple genes remains a major hurdle, and understanding the effects of gene expression kinetics on chronic wound healing outcomes will remain critical to developing translatable therapeutics. Continued advances in chronic wound healing therapeutics are needed to improve patient outcomes and reduce personal, financial, and societal burden. Innovations will be required in both nonviral gene delivery methods and bulk materials for encapsulating gene carriers, with a specific focus on multigene delivery systems with distinct release and expression kinetics for individual GFs.

## AUTHOR CONTRIBUTIONS

**James A. Mullin:** Conceptualization (equal); writing – original draft (lead); writing – review and editing (supporting). **Erfan Rahmani:** Writing – original draft (supporting); writing – review and editing (supporting). **Kristi L. Kiick:** Conceptualization (equal); funding acquisition (equal); project administration (equal); writing – review and editing (equal). **Millicent O. Sullivan:** Conceptualization (equal); funding acquisition (equal); project administration (equal); writing – review and editing (equal).

## CONFLICT OF INTEREST STATEMENT

The authors declare no competing financial interest.

## Data Availability

No new data were generated or analyzed for this manuscript.
